# Digitalising the Age-Friendly City: Insights from Participatory Action Research

**DOI:** 10.3390/ijerph17218281

**Published:** 2020-11-09

**Authors:** Arlind Reuter, Jennifer Liddle, Thomas Scharf

**Affiliations:** 1 Open Lab, Newcastle University, Newcastle upon Tyne NE4 5TG, UK; jennifer.liddle@newcastle.ac.uk; 2 Population Health Sciences Institute, Newcastle University, Newcastle upon Tyne NE2 4AX, UK; thomas.scharf@newcastle.ac.uk

**Keywords:** ageing, digital citizenship, age-friendliness, urbanisation, participatory action research

## Abstract

The World Health Organization’s age-friendly city initiative emerged as a response to the intersecting global trends of population ageing and urbanisation. However, a third global trend—digitalisation—has largely been overlooked in research and policy making relating to age-friendly cities and communities. Within the context of a general shift towards online civic participatory activities, this article explores older adults’ digital citizenship in an age-friendly city in the North of England. Drawing on interviews, observations and field notes from design workshops as part of an ongoing participatory action research project, we consider two key questions. First, how does an age-friendly city stakeholder organisation of older adults make use of digital technologies in order to provide digital information and communications? Second, what is the potential of digital audio to increase civic participation in later life and local engagement with age-friendly issues? Our analysis focuses on two domains of the World Health Organization’s age-friendly city framework: Communication and information and civic participation. First, we report on the stakeholder organisation’s efforts to re-design their digital newsletter in order to provide information and communications to older residents about local work on ageing projects. We then outline the organisation’s efforts, in a public setting, to engage with digital audio as a way to increase the participation of older residents with age-friendly topics. We conclude by suggesting the need to re-frame the role of digital technologies within the age-friendly city, broadening the scope from accessibility towards enhancing digital citizenship opportunities.

## 1. Introduction

The World Health Organization (WHO) age-friendly city (AFC) initiative emerged in 2005 as a response to the intersecting global trends of population ageing and urbanisation [1]. Supporting cities to become more age-friendly by promoting active ageing and creating accessible and inclusive city structures, the AFC initiative has since become a global program that has also been extended to include rural communities [2]. While some countries, such as Ireland [3], have established national age-friendly initiatives, others have created national networks that seek to harness learning and share best practice between age-friendly cities and communities. In the United Kingdom (UK), 41 cities, towns and counties currently belong to a network of age-friendly communities [4]. Rooted in the discourse on healthy and active ageing, age-friendly cities aim to provide a space to promote and maintain physical and mental health across the life course [2]. The AFC initiative is based on a framework that emerged from an empirical project involving focus groups of older people in cities around the world. Participants identified the following eight domains as important: Social participation; communication and information; civic participation and employment; housing; transportation; community support and health services; outdoor spaces and buildings; and respect and social inclusion [5]. These domains comprise core features that can be used to assess the relative age-friendliness of different cities and communities [1]. Despite the checklist format developed by the WHO to capture these core features, some researchers regard it as more helpful to frame age-friendliness as an ongoing process and commitment to improve the physical and social environment of a city in response to the challenges and opportunities arising from demographic ageing [2,6]. The process to become an age-friendly city or community is based on a cyclical model for continuous improvement that is driven by older people themselves. It involves four stages: Engage and understand; plan; act; and measure [2]. While the age-friendly concept can be implemented in different ways across the domains, underlying the framework is a life-course perspective that acknowledges diversity at all life stages and promotes citizen contributions to all areas of community life [1].

Accompanying the global age-friendly agenda has been the rapidly increasing digitalisation of cities and communities. While there is a growing evidence base that highlights the need to explore digital aspects of later life, including, for example, re-framing stereotypes on ageing and information and communication technologies (ICT) use [7,8] or using the internet for civic or social participatory purposes in later life [9,10], the intersections between the WHO age-friendly domains and widespread digitalisation remain under-explored within the age-friendly city framework [11]. Within the domain of communication and information, for example, the WHO AFC checklist includes a brief mention of ICTs, but the information is limited to the accessibility of certain technological devices, such as mobile phones or public computers. Digital technologies are entirely absent from the civic participation domain of the checklist, thus ignoring the increasing role played by digital approaches in different forms of citizen engagement.

In this paper, we respond to the challenge of integrating digitalisation trends into existing debates about demographic ageing and urbanisation that underpin the global age-friendly initiative. Our approach entails exploring and positioning the concept of digital citizenship within the context of a medium-sized city in the North of England that belongs to the UK network of age-friendly communities. Working within the AFC domains of communication and information and civic participation and drawing on interview data, observations and field notes from a participatory action research project involving an AFC stakeholder organisation of older adults, we add to the current understanding of age-friendly cities and communities by providing practical examples of digital citizenship in later life. We seek to highlight the value of extending the WHO’s age-friendly initiative beyond a narrow focus on digital accessibility and inclusion issues to incorporate the growing importance of digital citizenship in later life and the potential of digital approaches to facilitate active ageing by reducing the risks of digital exclusion faced by ageing adults.

## 2. Background

### Digital Citizenship

Within the discourse on age-friendly cities and civic engagement lies the concept of ‘urban citizenship’, which recognises older adults as active agents who have a right to make full use of the city [12]. In their ‘Manifesto for the Age-Friendly Movement’, Buffel et al. argue that the concept of the ‘right to the city’ includes appropriating urban spaces, participating in decision making and influencing strategies of urban planning and regeneration. Urban spaces are characterised by growing inequalities within and between cities, including a trend towards increasing age-segregation. Preventing the exclusion of older people from participation in age-friendly initiatives, especially people belonging to disadvantaged groups, involves ensuring that all older people have opportunities to exercise their right to have their voice heard [13]. In terms of older adults’ civic involvement at a local level, Buffel et al. highlight the paradox of neighbourhood participation: Even though older adults spend more time than younger people within the immediate neighbourhood of their homes, they are often the least, or last to be, engaged in civic decision making [13]. Moreover, in increasingly digitalised cities and communities, there is a fundamental need to reconsider what ‘urban citizenship’ entails and to reframe the potential role to be played by older adults as ‘digital citizens’ in shaping age-friendly cities and communities.

Whilst ICTs have been acknowledged as resources that might support older adults’ involvement in local decision making [13], approaches are often solutionist [8]. A consideration of the role of digital technologies is also lacking within the WHO AFC framework [11], prompting responses to address this gap. For example, Marston and van Hoof have proposed a revision to the WHO framework to include technology as part of a smart age-friendly ecosystem that supports older adults’ daily lives through ubiquitous and assistive technologies [11]. More recently, a revised definition of age-friendliness has emphasised the role of digital technologies within age-friendly settings:

*Underpinned by a commitment to respect and social inclusion, an age-friendly community is engaged in a strategic and ongoing process to facilitate active ageing by optimising the community’s physical, social and digital environments and its supporting infrastructure*

*[14] (p. 19).*

Adopting the above definition of age-friendliness to reflect the crucial role of the digital environment as a facilitator of active ageing in age-friendly cities and communities, our research explores the use of digital technologies for a civic purpose, which is a concept that has been under-theorised and largely overlooked in relation to the AFC. We want to expand this discourse on technology within the age-friendly city by highlighting the concept of urban citizenship and its digital aspects, proposing a shift from technology as a solution in the age-friendly city towards a focus on the digital civic contributions of older adults. Digital citizenship is broadly defined as the ability to participate digitally within society on a regular basis [15]. In research, the subject of Digital Civics has been considered in the discipline of Human–Computer Interaction (HCI) [16,17]. It seeks to understand how technologies can be designed in the context of civic interactions and experiences. This can be done by turning to participatory systems that enable citizen engagement and the creation of counter narratives in a relational rather than transactional way [18], thus opening up a space for activism [19]. By recognising and acknowledging the effects of the digital divide, a digital civics research approach supports (marginalised) communities in co-producing political thinking, action and dialogue through relational technologies [16].

The focus on digital civics is especially important within the context of demographic ageing. Older adults have been shown to be at a heightened risk of exclusion from civic participatory activities [20]—a problem which is exacerbated by the digital divide [21]. However, as opportunities for civic participation increasingly shift from offline to online (e.g., creating and signing petitions online or engaging with local government services), more older adults are using technological tools in order to participate in digital civic activities [22]. Indeed, the number of older adults who are using the internet has been increasing in recent years and 83% of adults aged 65–74 and 47% of adults aged 75 and over use the internet in the UK [23]. In addition, 98% of UK premises have access to broadband and internet [24]. Buffel et al. propose that “information and communication technologies may support the involvement of older residents in navigating and designing their environment” [11] (p. 281). This aligns with the recent movement within the HCI community towards recognising forms of older adults’ engagement with civic technologies in community settings [25,26]. Regarding the design of participatory systems or technologies, the field of social computing has progressed from designing for older adults [27] towards involving older users in the design of services [28] and reflecting on co-design methodologies, specifically within the context of ageing research [29,30,31]. An example of a co-design process was provided by Clarke et al., who used participatory media to explore older adults’ engagement with the city in the context of urban planning [25,32]. In particular, the interdisciplinary design team considered “how technology can support documentation and re-envisioning of the age-friendly city of the future” [30] (p. 2). The study outlines how technology could be a mediating factor in urban planning contexts, facilitating discussions between local councils and older citizens. The contribution of Clarke et al. is important in demonstrating that digital media in particular can support older adults in making active contributions within their neighbourhood, highlighting key concerns for ageing beyond accessibility.

The COVID-19 pandemic has reinforced the imperative for the active participation of older adults in public debates. Given ongoing digitalisation processes, a stronger focus on “technology” as a key feature of the age-friendly city concept has become inevitable. However, the pandemic has focused attention on the need for individuals to be online in order to participate fully in social and civic life. The pandemic has also emphasised the importance of digital activity among older adults, with the digital inclusion of older adults gaining greater attention in government initiatives and within third sector organisations [33,34]. To date, local council digital inclusion strategies, in the UK at least, predominantly adopt a needs-based framework that addresses factors of access and accessibility (e.g., the ability to connect to the internet or “knowing where to start online”) [34]. We distinguish between this form of digital inclusion and the concept of digital participation, which encompasses the active use and contributions of older adults in the digital space [35]. Indeed, older adults are increasingly participating in and actively contributing to digital spaces by creating their own content in the form of blogs, videos and radio/audio broadcasts [10,36,37]. Recent studies have examined older adults’ content creation [9,10,38,39], suggesting that one of the key motivations for producing content lies in “being advocates for older people” [9]. While such developments emphasise the civic purpose of older adults’ digital participation, this idea has yet to be directly picked up by government or age-friendly initiatives.

The body of work described above highlights the current, somewhat limited, discourse on technology and its role in supporting citizenship in age-friendly cities and communities. Given the pace with which the digitalisation of society is progressing, there is a need for a more differentiated perspective on age-friendly initiatives that can benefit from insights drawn from different scientific fields. Our work sits at the intersection of research on age-friendly environments within the field of social and environmental gerontology and related work undertaken in HCI. While the WHO age-friendly program has operated in an increasingly digitalised environment, linked policies and initiatives have, to date, not sufficiently addressed the concept of digital citizenship. Digital inclusion strategies have focused on access and accessibility, neglecting a consideration of older adults as active digital contributors. In this paper, we explore and position the concept of digital citizenship within the context of a medium-sized age-friendly city in the North of England by examining the civic use of digital technologies. We provide detailed qualitative insights into how a community organisation of older adults engages with digital communications and digital audio in order to facilitate and promote their engagement in the process of making their city more age-friendly. More specifically, we consider how the organisation operates within two of the eight domains identified in the WHO AFC framework: Communication and information and civic participation. As a result, our research seeks to contribute to discourse around digital participation and digital citizenship, making the case for the central role of these activities in age-friendly city and community programs. We present the methods and findings of our study in the following sections.

## 3. Methods

Our paper draws on an empirical study, which set out to explore digital citizenship of older adults within the context of a medium-sized city in the North of England that belongs to the UK network of age-friendly communities. As with other medium-sized cities in the UK and internationally participating in the WHO AFC program, the city is actively engaged in responding to the combined opportunities and challenges associated with demographic ageing and urbanisation [40,41]. Working with an AFC stakeholder organisation of older adults in the city, and reflecting where we saw evidence gaps in existing literature on age-friendly cities and communities, we sought to respond to two key research questions:

1. How can stakeholder organisations of older adults best exploit digital technologies to provide digital information and communications to support age-friendly city initiatives?
2. What is the potential of digital audio to promote civic participation in later life and to facilitate public engagement with age-friendly issues?

We draw on interviews, observations and field notes from design meetings collected as part of an ongoing participatory action research project, working collaboratively with an AFC stakeholder organisation. The project received institutional ethical approval (Ref. 8659/2018) and our work is being conducted in line with the ethical guidelines of the British Society of Gerontology [42], valuing a collaborative research process guided by the collaborators’ experience. Pseudonyms are used to refer to all collaborators named in this paper.

### 3.1. Research Context

In early 2018, we (an interdisciplinary research team of HCI and social gerontology researchers) approached an organisation of older adults in the North of England, whose members are heavily involved in shaping the discourse around age and ageing in their city at a social and political level. The organisation has been a key stakeholder in the city’s AFC group since the group was established. We engaged with the organisation using participatory action research (PAR), which is a highly contextual and localised approach to community-based research that typically aims to generate sustainable social change [43]. Reflecting its democratic and collaborative ethos, PAR requires constant communication between researchers and collaborators working with a cycle of “planning, acting and reflecting” [43]. Having engaged with the wider organisation in line with PAR principles, this paper reports on a specific collaboration with the organisation’s communications team leaders, who self-selected and approached the authors for this project. We implemented the PAR process by using co-design methods and interviews at several stages throughout the project, as outlined below. In this paper, we report on one specific component of the broader PAR project, which is investigating how the organisation and its members engage with digital technologies in order to increase older adults’ civic participation in age-friendly work across the city. Reflecting a commitment to promoting age-friendliness in multiple spheres of city life, the collaborating organisation engages in regular campaigning. In order to increase the impact of their activities, they use both digital and non-digital strategies to engage with older people across the city. In this paper, in line with the research questions specified above, we report on how the organisation works digitally within two of the WHO’s AFC domains: (1) Information and communication, by communicating age-friendly work digitally, and (2) civic participation, by using digital audio to engage citizens in public spaces on the age-friendly agenda.

### 3.2. Working within the Information and Communication Domain

One element of the organisation’s digital engagement takes place through its digital communications. Throughout the PAR project, it became clear that members of the organisation wished to restructure some of their digital output, in particular, their online newsletter. The newsletter had previously been prepared using Microsoft Word and circulated as a pdf file attached to an e-mail. In order to allow a more collaborative workflow and, more importantly, gather, for the first time, statistical information about user engagement, the organisation’s communications team wished to shift their newsletter to the Mailchimp platform instead. Mailchimp allows content creators to manage mailing lists, newsletters and other digital content, whilst also providing users with insights into how content is accessed by its audience. In response to our first research question, we now report on the design process of shifting the organisation’s newsletter to Mailchimp with the aim of exploring digital communications within the AFC context.

#### Research Methods and Analysis

Our analysis of this design process draws on two types of data (see Table 1). First, four interviews were conducted with the organisation’s three communication leads (Sarah, Iris and Dora, who are older people themselves). Interviews took place at different stages throughout the project. Second, observations and field notes from nine design meetings (average duration of 90 min) were made throughout the year 2019. The semi-structured interviews openly explored the organisation’s information and communications work, as well as asked specific questions about the organisation’s digital engagement within the AFC context (e.g., “What is the commitment of the AFC stakeholders regarding digital technology?”). All interviews were audio recorded and transcribed verbatim. The design meetings with the purpose of re-designing the organisation’s e-mail newsletter were attended by A.R., a professional content creator and two of the organisation’s communications leads (Iris and Dora). Field notes were taken contemporaneously and the researchers added further observations after each meeting as part of their research diaries. The design meetings provided an opportunity to collaboratively explore the organisation’s existing media output and actively support the design of their e-mail newsletter in terms of content, functionality and layout. This design process was both guided by specific milestones that were important to the organisation (e.g., initiating a name change for the newsletter), and unstructured, as an open space for feedback, suggestions and ideas (e.g., personal challenges with the technology). We tried to create an informal atmosphere, as the meetings took place at the research facility. The aim was to signal that sufficient space and time were available to discuss any technical topics that emerged, despite being located in a school of computing. We provided tangible design materials, such as paper and pens, as well as tablets and laptops, to demonstrate ideas on a big TV screen.

We used reflexive thematic analysis [44] to analyse the interviews and field notes. Thematic analysis is a way to make patterns of shared meaning visible within a dataset, acknowledging the active role of the researcher as part of this process [44]. It is a flexible approach to qualitative data analysis and can be used within different theoretical frameworks [45]. We approach this analysis from a critical realist perspective, acknowledging the experience of our interviewees whilst at the same time contextualising these experiences in how they are shaped within a broader social context [46]; in our case, the age-friendly city’s dynamics. We carried out the analysis in two stages. First, A.R. applied codes to the transcribed interviews in NVivo 12 software (QSR international, Burlington, MA, USA). The first stage of coding was carried out inductively to explore the transcript in its entity. In stage two, we repeated the coding process with a deductive focus to hone in on digital aspects whilst also integrating the field notes. All codes were discussed and the theme we derived in relation to the information and communication AFC domain is “communicating age-friendly work digitally”. The theme is based on codes that consider audience engagement, collaborative work and the intersection of digital and non-digital aspects, all of which are presented in the findings section.

### 3.3. Working within the Civic Participation Domain

Our second research question aims to consider the role of digital technologies within the AFC civic participation domain. We explored this domain in collaboration with the same AFC stakeholder organisation, given the organisation’s aim to advocate for older persons and their rights. Throughout 2019, for one day each month, the organisation was able to secure a unit in the city’s centrally-located, historic covered market, in order to promote themselves and their work. The market is a popular destination for shopping and a space for intergenerational encounters. As the venue was offered to the organisation free of charge, no alternative was considered. The organisation’s first day at the market generated only a low level of public interest. Reflecting the principles underlying the ongoing PAR project, one member of the organisation’s communications team, Sarah, felt able to subsequently initiate two exploratory meetings with A.R. to discuss the potential of using digital media as a method to increase public engagement.

#### Research Methods and Analysis

The exploratory meetings were conducted as a combination of informal discussions and structured ideation. The sessions were spent exploring the goals of using digital technology as part of the organisation’s engagement sessions at the market in an informal discussion, namely (1) engaging citizens in age-friendly work and (2) demonstrating impact (as outlined in the results section). We subsequently developed a digital engagement strategy for the organisation’s market unit.

**Figure 1:**
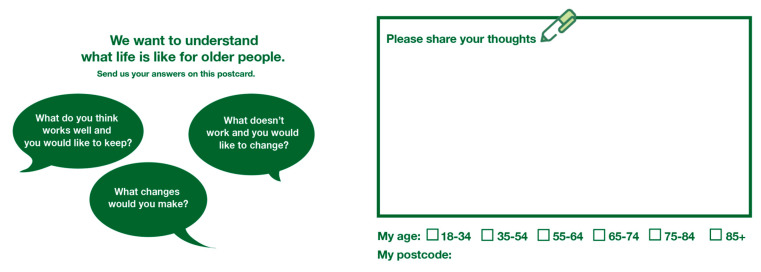
Front and back of feedback postcard.

The organisation had previously completed a listening exercise in which they sought older people’s opinions on various issues across the city, presenting the results as a written report. The members now voiced interest in replicating this listening exercise in a digital form. A.R. adopted a ‘user scenarios’ [47] approach as an ideation technique to co-design a digital engagement strategy to generate interactions in the market. Talking through different imaginary user scenarios, such as “What do people think when they pass the market unit?”, “Why would people want to give their opinion?” or “How can we attract the interest of older people who are shopping in the market?”, Sarah decided that using audio was an interesting and privacy-aware way of engaging people through technology, as well as capturing voices on a range of age-friendly topics. Despite wanting to create a more tangible evidence base on how older people respond to certain topics, the organisation wanted the audio to predominantly “spark discussions, rather than being too rigorous or methodological” (Sarah), emphasising the importance of using technology-based designs as a scoping tool to elicit topics of interest. As a topic for their listening exercise, the organisation was especially keen to explore opinions on intergenerational cohesion within the city. Through iterations of the ideation approach described above, we developed the idea to collaboratively create recordings of intergenerational provocative discussion starters. Six younger people and six older people were asked to respond to statements such as “I am proud of my generation”, “I am grateful to the older generation” or “I am annoyed with my generation because…”. The responses were audio recorded. We used the organisation’s iPad to play these provocative statements. Even though we planned to use the audio recordings at the market as a playful way to engage people in discussions, we also wanted to provide a non-digital option to gather feedback. As an alternative for people who would prefer not to be audio recorded, we created small postcards that would encourage people to express their opinions (see Figure 1).

We analysed the findings from the ideation activities, as well as from the actual process of using digital audio in the market setting. A.R. analysed the field notes using reflexive thematic analysis, as previously described in the research methods of Section 3.2.

## 4. Findings

We now report our findings on how a specific AFC stakeholder organisation operating in a medium-sized city in the North of England works digitally within the two AFC domains addressed as part of our research questions. We start with the communication and information domain, before moving on to the civic participation domain. These two aspects of the organisation’s digital interactions provide an example of how the concept of digital citizenship is already addressed within an AFC. First, based on interview data and the re-design of their online newsletter, we report on how the organisation communicates their age-friendly work in a digital form. Second, drawing on results from the organisation’s market engagement, we present the results on their use of digital audio as a way to engage older people with age-friendly topics in public spaces.

### 4.1. Communicating Age-Friendly Work Digitally

In this section, we present findings on how the organisation works towards communicating age-friendly work digitally regarding the AFC information and communication domain. Thematic analysis (TA) of the fieldnotes and interviews in relation to the organisation’s communications and, especially, the re-design of their online newsletter suggested that the organisation perceived the potential to reach and engage a wider audience as the main benefit to be derived from engaging with digital communications. We present an overview of the themes and how they were derived in Table 2.

Building on the organisation’s previous discussions about the need to offer digital communications to older people across the city, the team emphasised the importance of using online communications in order to reach older adults, in particular, people in their 50s. Approximately 15 years ago, as part of the city’s age-friendly efforts, the organisation was involved in developing an online platform to provide information for older adults. Sarah and other members of the AFC initiative advocated for the platform to be developed digitally at the time:

*“When we did that back in 2006, 2007, we got a huge amount of flak because people said that older people don’t go online. Thanks very much to a colleague of mine, who really said, ‘No, we’ve got to do this as a digital platform, this has got to be online’ we really stuck to our guns. So,* [the website] *is a key information resource for people in the city, but alongside that, we then tried in a small way, I guess, to create opportunities for older people to improve their digital skills.”*

*(Sarah)*

The struggle that Sarah and other older people’s advocates faced when suggesting a digital platform highlights a lack of awareness relating to the topics of technology and ageing at that time. Sarah emphasises that using a digital platform can be an inclusive way to communicate age-friendly work to a wider audience, not just older adults themselves. The organisation aims to use their online communications and, in particular, their digital newsletter, which is mainly produced by the volunteers Iris and Dora, to engage older people across the city. The organisation wished to change the format of their newsletter from a pdf document to electronic content managed within a platform (Mailchimp) that merges design tools with audience management, in order to increase the marketing effectiveness. During the process of changing the format, we developed a structure within the newsletter that first emphasises the organisation’s achievements within the AFC and other ongoing activities, before providing general information on activities across the city. This structural design choice was made by the organisation in order to heighten awareness of their work as older people’s advocates across the city.

The development of the Mailchimp newsletter was driven by the desire for obtaining insight into audience statistics. Indeed, within a few months following the format shift, the organisation gained feedback as to how their newsletter was received by the public:

*Iris: “We also know, from looking at the analysis, what’s happening with it, which we didn’t know from Word. We knew nothing from Word at all, we just knew how many people were getting it. So, it’s about a 50% opening rate.”*

*Dora: “I can give you the facts and figures. The first one we did was on 4th October and it went out to 569 people, had a 45% open rate and an 8.3% click rate.”*

This shows that both Iris and Dora are keen to stay updated with their audience statistics, tracking their opening rates and trying to understand user experience and engagement with the content that they distribute. The organisation uses the free version of Mailchimp rather than the paid options, which would allow for more detailed statistical insights. However, for the time being, they emphasise that having at least some limited insights is more useful than having none, as was the case when producing the newsletter in its earlier format. Despite this, the team continues to be interested in establishing correlations between specific topics addressed in the newsletters and user engagement:

*“Because it would be helpful if we knew what was in the highest rating clicks, rather than look at the ones where… you know, so we don’t have to do every one, but if we get a really positive reaction to a particular* [newsletter]*, what was it in that* [newsletter] *that really attracted people’s attention. That would be valuable.”*

*(Dora)*

This highlights the group’s intention to tailor the content of the digital newsletter based on their audience’s interests and to further analyse the impact of their activism. In addition to this digital feedback on audience engagement, the team had also received personal feedback from people across the city. However, despite originally aiming their newsletter at older individuals, the team noticed that other local organisations were also making use of the information presented in the newsletter:

*“We always knew how many people it was going to. We have picked up more people, but they’re organisations, rather than individuals. So, we find a lot of organisations are using what we’re putting out, which is an interesting thing.”*

*(Iris)*

This engagement with the newsletter outside of their own membership shows the power of digital communications within a network of AFC stakeholders. Our findings suggest that by creating a cycle of information, in which different organisations promote each other, age-friendly topics and events can gain much wider attention. This heightened engagement through cross-promotion also reflects the importance of being strategic about promoting the work carried out as part of the AFC initiative. When the city initially joined the WHO’s global age-friendly initiative, Sarah, as a stakeholder within the AFC, *“tried quite hard to get a communications strategy going* […] *but ended up not pursuing it, which probably was a very bad mistake.”* Our findings highlight that something as complex as a communications strategy cannot easily be organised and executed by an individual person and requires specific digital communications skills, insight into the AFC group and collaboration between the stakeholder organisations, as well as a time commitment. Time constraints and the need to increase the production efficiency served as additional reasons to re-design the organisation’s newsletter:

*“I would like us to sit down together with the newsletter and email page and have a look at how you use it and how I use it. Because I think that I don’t use it in the most efficient way.”*

*(Iris, Interview 1)*

Throughout the design meetings, Dora and Iris agreed that, by working collaboratively as a team, the organisation’s media output could be maximised. However, despite developing the concept and design of the newsletter together with the aim of increasing collaborative work, the interview in February 2020 revealed that the team members ended up taking the production in turns. We asked them to outline their reasons for continuing to work individually and Dora said:

*“From my point of view, the commitment with that is quite different because, if you were both doing it at the same time, you’re not actually having a break from it. I mean, I might put something in, or Iris might put something in and I might go, “That’s not suitable. Take it out.” So, there’s the different interest, different… and Sarah could come along and do something completely different. I’m happy with doing it two, four, however many issues and then having a break and it gives you time to think of other things as well and to read it when somebody else has done it and see, pick up different things.”*

*(Dora, Interview 4)*

This reflects the considerable time commitment that is associated with digital communications and the importance of being independent in the editorial process. Viewing the production of the newsletter as a process towards collaborative work, rather than an immediate new state of working, reflects the busy nature of the organisation’s team of volunteers, who are learning new digital skills in order to advocate for their organisation online. In addition to working towards collaboration within their digital outputs, the communications team was mindful of creating face-to-face and non-digital interactions:

*“So, some of it needs to be face-to-face, we still like that, we like face-to-face, we like paper, we like all of that, we’ve got to have, but, actually, we also need this other dimension, which is both about trying to get broader engagement, but, also, about how we present ourselves to the world.”*

*(Sarah)*

Valuing both face-to-face interaction and digital engagement is a relevant factor for Sarah, as the group’s organiser. Being connected physically is crucial for running a network, such as the AFC group and its stakeholders, whilst using digital means can connect the group to the wider public and showcase the wider collaborations within the AFC group.

To summarise this component of our findings, we have outlined the importance of communicating age-friendly work digitally in terms of reaching wider audiences, showcasing collaborations across the city and creating efficient workflows. We have also reported on the importance of balancing digital and non-digital interactions within the age-friendly city. The next section discusses an example of digital work organised by the collaborating organisation, using digital audio to increase public engagement on age-related topics.

### 4.2. Using Digital Audio to Increase Civic Participation

In this section, we present findings from the organisation’s digital work within the AFC domain of civic participation. We describe two themes (see Table 3), which outline why the organisation chose to experiment with digital audio as a way of heightening their civic impact: (1) The opportunity to engage citizens more widely, and (2) as a way to demonstrate the impact. We then report on findings from the activity of using digital audio in the local market hall.

In April 2019, the organisation and A.R. used the digital audio statements as a way to engage older citizens on age-friendly and civic issues in the local market hall. As the first few exhibition days in the market unit were characterised by a notable lack of interest from passers-by, Sarah reflected that the issue lay in the way they were trying to reach their audience:

*“Why is it [that people] are not interested? I’m sure they are, but we’re certainly not reaching them. The younger cohort. People in their 60s.”*

*(Sarah)*

She perceived, in particular, younger cohorts of older adults as being more tech-savvy, concluding that it is important to use digital channels in order to promote the organisation’s political and civic work. Using digital audio as a way to capture people’s attention in the market unit and to invite feedback was seen as an opportunity to engage passers-by in deeper discussions (as opposed to paper-based leaflets that are handed out) and to learn more about residents’ opinions, in order to reflect them in their activist work. Technology was also seen as a novel and interesting way to attract people to visit the unit, in particular, people who might not previously have heard about the organisation or the AFC work.

The second reason that underlay the organisation’s motivation to engage with digital audio was the possibility to demonstrate the impact. As a third sector organisation, the group is partly dependent on external funding for their projects. As an advocacy organisation, “evidence” is often deemed necessary to have an influence on policy development. Presenting more tangible and creative insights into older residents’ experiences in a digital format compared to a written report can provide a starting point for discussions on age-friendliness in more formal political settings. Sarah assumed that involvement with digital audio could transform the reputation of the AFC stakeholder organisation from *“inward looking to knowing what a range of older people in the city are actually interested in [by listening to them]”*, thus being more responsive to older citizens. This reflects the potential of digital audio to be a communications tool within the AFC that supports bottom-up citizen engagement. Using digital audio within the local market was seen as an opportunity to capture diverse opinions from older local citizens.

**Figure 2:**
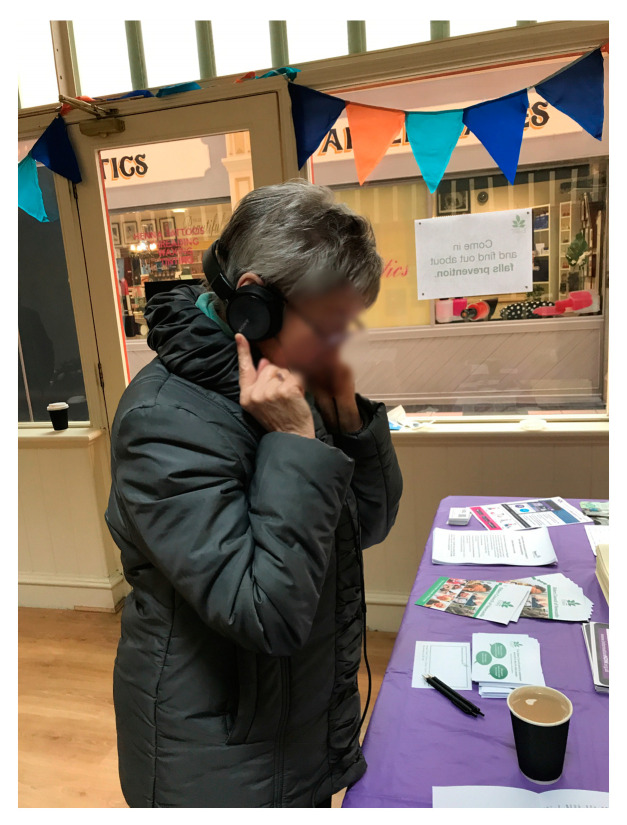
Listening to the audio statements.

Over a duration of four hours, members of the organisation used the iPad to play the pre-recorded statements by older and younger people to passers-by (see Figure 2). A.R.’s field notes recorded that “many members of the organisation joined the team on the day, eager to promote their organisation’s work”. The setup of the iPad with the headphones caught many people’s interest as they were passing by. The organisation’s members actively approached and prompted people in the market to join them and listen to the recordings. Indeed, over the course of the morning, around 15 people listened and engaged with the group as a response. However, despite this heightened level of interest, only four people were willing to record their own responses. These responses were on average 1.26 min long and, despite our prompts to share opinions on intergenerational cohesion or raise local issues, the responses included a variety of topics: Food banks and social inequalities within the city; personal health issues; an interest in the history of the city; and a general political critique. Indeed, many of the people who chose not to be recorded were triggered by the intergenerational provocations to voice their concerns about or agreement with national issues. The experience showed that despite prompting for a certain topic, using audio sparked highly emotive discussions on a variety of topics and, in particular, on the wider political landscape. As an evaluation, the organisation and researchers jointly agreed that the approach of using audio as a way to collect evidence on a specific topic in a public space had its limitations. These were mainly due to people’s concerns or shyness about being recorded and the wide variety of unanticipated topics raised by members of the public. We concluded that the pre-recorded statements needed to be refined and less provocative, in order to generate responses on topics that are relevant to the organisation and that can be used specifically for advocating for age-friendly work within the city. However, the intervention was not followed up directly, as the organisation decided to pursue a different strategy for their market unit in the following months.

Additionally, the organisation and authors prepared non-digital materials (see Figure 1), which most people who engaged with the organisation’s members at the market unit also filled out. Approximately 15 postcards were collected, outlining different issues of relevance to AFC domains, such as parks and green spaces, historic tours and personal health issues. Overall, the researchers questioned whether the activity aimed to engage people in too many different things at once (listening to audio, handing out leaflets, and filling in postcards), which led to a loss of focus on the information that the organisation wanted to capture. Nonetheless, the range of formats provided numerous opportunities for engagement and resulted in an overall heightened engagement and interest compared to the organisation’s initial experience at the market.

To summarise, the AFC stakeholder organisation used digital audio provocations as a way to spark discussions on intergenerational cohesion and capture older residents’ voices on other age-friendly topics. The use of a very basic digital technology (iPad with audio) proved to be an effective way to engage people and attract people’s attention to the organisation’s market unit. However, the project provoked discussions on a wide range of topics, not necessarily reflecting age-friendliness, but nonetheless giving an opportunity to older adults to share with an AFC stakeholder what was going on in their lives. In addition, privacy concerns and the shyness of participants were underestimated in our design, resulting in an unwillingness of members of the public to be audio recorded. We concluded that, due to those reasons, audio might be a way to spark discussions, but despite its benefits to promote civic participation, it might not be the optimal route to capturing age-friendly interests in contexts such as the market setting.

## 5. Discussion

In this paper, we have explored different aspects of digital citizenship within the age-friendly city. Our research questions investigated (1) how organisations of older adults can best exploit digital technologies to provide digital information and communications to support age-friendly city initiatives, and (2) the potential of digital audio to promote civic participation in later life and to facilitate public engagement with age-friendly issues. We responded to these questions by illustrating how one AFC stakeholder organisation of older adults works within the age-friendly domains of communication and information and civic participation. Reporting on those two domains, we have outlined how the organisation makes use of digital communications in order to promote age-friendly work across the city. Additionally, we have reported on how the organisation used digital audio as a way to capture older people’s voices at the local market as an effort to engage older citizens and provide evidence for older adults’ civic participatory work.

Before discussing key insights of our research, such as the need to create digital visibility around age-friendly projects and to consider the importance of digital citizenship for older adults by looking at civic opportunities in online and offline spaces, it is useful to address some of the limitations of our approach.

### 5.1. Limitations

Our qualitative and participatory methods give insight into the practices of one particular age-friendly city in the North of England. Facing high inequalities in the wider region, age-friendly initiatives are highly dependent on the goodwill of local activists and politicians as other political issues have been prioritised over the years. Whilst our findings are limited to this particular local context and constrained by the obvious limitations of a small-scale empirical study, this is also a feature of other work on age-friendly cities and communities [48]. Moreover, the points raised in regard to digital citizenship as one aspect of promoting age-friendliness and, in particular, the importance of using digital media as a way to achieve this, are likely to be of wider interest to researchers and policy stakeholders engaged in AFC work in other geographic locations. Whilst some age-friendly cities have created an “AFC brand” for themselves and work with professional communications strategies, we, in contrast, report on how older adults themselves are acting in ways that can advocate for a stronger focus on age-friendliness in their cities and communities. As a result, we believe that our approach and findings are of importance to other age-friendly cities and communities who might not have the financial resources to advocate for AFC work professionally, but rely on older activists to communicate and engage citizens in age-friendly actions [49,50].

### 5.2. Creating Digital Visibility of Age-Friendliness

Throughout the research reported in this paper, we noticed that digital engagement was predominantly perceived and used as a tool to discuss social issues or collect “evidence” for opinions on political issues, therefore offering a way to uncover issues that are of importance to local older residents. Returning to the idea expressed by Buffel et al. in their manifesto for age-friendly cities of having a ‘right to the city’ [12], we associate this right with the opportunity to be able to use digital technologies as a means to achieve civic expression and a mechanism to actively contribute to civic debates in later life. We found that communicating AFC work through digital means is an important factor in how age-friendly a city is perceived to be by a public audience. Our research, conducted in a single location, highlights that cities which have not invested in a professional communications strategy might therefore be perceived as being less age-friendly than those whose work is more digitally visible, despite ongoing efforts and a great amount of activity around the topic. Echoing the findings of Clarke et al. on using digital media as a way to advocate for older people’s issues at a neighbourhood planning event [25], we also established that using digital audio recordings to create evidence for political purposes was not always successful. However, while creative approaches may not necessarily produce measurable AFC outcomes, our study suggests that using digital audio may be useful as a conversational prompt. This points to the potential of using digital technologies as a creative way to engage people in discussions around the AFC and to initiate the dialogue with older adults that is necessary to underpin the development of age-friendly policies and practices in the future.

### 5.3. Considering Digital Citizenship in Later Life

As a main contribution of this study, we highlight the importance of considering the intersection between digital technologies and citizenship in later life as a pathway to supporting age-friendly cities and communities. Technology is often incorporated into cities with a focus on accessibility, supporting older adults’ daily lives through smart assistive technology [11]. Our study extends this focus by contributing to the discourse on digital and civic forms of participation within the context of a burgeoning, global age-friendly movement. In the literature review, we identified a push within the field of Human–Computer Interaction towards recognising older adults’ diversity [8] and towards evaluating how older adults make use of digital technologies within their community settings [26]. We add to this line of argument by blending gerontological and HCI research approaches and by highlighting the civic opportunities that the use of digital technologies offers as part of an AFC initiative. Indeed, as far as we are aware, based on our review of existing literature and policy documents, there are currently no guidelines in the public domain that would support older adults’ digital citizenship. Our particular focus has been on the importance of having a voice in later life and the need for citizens to be adequately informed about age-friendly topics. The research reported here emphasises the crucial need to re-frame the (rather limited) concept of digital inclusion and to focus instead on supporting the active digital participation of older adults, strengthening their digital citizenship. Supporting older adults’ digital participation, not only their inclusion, could be an asset for age-friendly city initiatives. Acknowledging a more active civic contribution of older adults in online spaces has the potential to challenge ageism in relation to digitalisation and expand the diversity of online discourse by supporting different voices to be heard. While the COVID-19 pandemic has been associated with an increase in older adults’ visibility in online spaces, we suggest that AFC initiatives should prioritise activities that support older adults in becoming more active in digital civic activities. Using a combination of digital and non-digital tools, such as supporting offline efforts to encourage civic participation with digital media, can be a way of creating spaces for meaningful civic participation.

## 6. Conclusions

The research presented in this paper explored digital citizenship within the age-friendly city context. It adds to key debates on technology and ageing by highlighting a need to include digital citizenship as a factor within discourse on the role of technology in the age-friendly city. We have shown that while digital communications are crucial for informing the public about age-friendly work, older adults often face specific challenges in creating those communications. We have also demonstrated that digital audio can be a creative way to engage the public in discussions on a range of age-friendly topics. These findings have implications for the delivery of age-friendly projects, highlighting a need to incorporate digital and non-digital elements.

Our work also points to an important research agenda that exists at the intersection of the global trends of ageing, urbanisation and digitalisation. There is ample scope for future research to consider how different digital technologies can support an increasingly diverse population of older adults in urban settings in engaging with the age-friendly cities and communities agenda. Adding a civic participatory element to the predominantly needs-based narrative on ageing and assistive technologies is key to achieving the objectives of the global age-friendly movement. In this context, it will be of particular interest to discover new and creative ways through which digital technologies can facilitate a dialogue with older residents and age-friendly city groups. Equally relevant are studies that explore how age-friendly initiatives can be supported further to heighten awareness for their work in digital spaces. This is an exciting research agenda that necessarily cuts across scientific disciplines, engages with multiple methods of enquiry, and benefits from close collaboration between researchers and citizens.

## Figures and Tables

**Table 1:** Interviews and design meetings timeline.

Members	Time	Topic
Iris and Dora	February 2019, Design Meeting 1	General ideas for the newsletter layout
Iris and Dora	February 2019, Design Meeting 2	Developing a Mailchimp concept to be presented to the wider organisation
Iris and Dora	March 2019, Design Meeting 3	Setup of Mailchimp account
Iris and Dora	March 2019, Design Meeting 4	Template setup and content layout
Iris and Dora	April 2019, Interview 1	Creating efficient workflows
Iris and Dora	April 2019, Design Meeting 5	Ideas for cover stories and name of newsletter
Iris, Dora and Sarah	May 2019, Interview 2 as part of Design Meeting 6	Re-design of newsletter to Mailchimp
Iris and Dora	September 2019, Design Meeting 7	Technical Advice
Sarah	September 2019, Design Meeting 8	Creation of mailing list and campaign
	October 2019: first digital Newsletter	
Iris	December 2019, Design Meeting 9	Increase audience engagement
Iris and Dora	February 2020, Interview 3	Evaluation of use of Mailchimp
Sarah	February 2020, Interview 4	Digital AFC and communications

**Table 2:** Themes, example codes and data from the thematic analysis (TA) exploring the digital newsletter.

Themes	Example Code	Data
Reaching wider audiences	Retrieving audience statistics	*It went out to 569 people, had a 45% open rate and an 8.3% click rate. (Dora)*
	Getting personal feedback	*There was a lovely comment yesterday. A lady had sent a message in. (Dora)*
Showcasing AFC collaborations	Networking organisations	*So, we find a lot of organisations are using what we’re putting out. (Iris)*
	Strategically distributing information between AFC members	*I’ve been at a meeting this morning and both the ladies that were there said ‘We read the articles and we share with our members’ (Dora)*
Creating efficient workflows	Committing time	*It’s capacity timing, isn’t it. That’s the problem with that. (Iris)*
	Learning from each other	*I would like us to sit down together with the newsletter and email page and have a look at how you use it and how I use it. (Iris)*
Balancing digital and non-digital interactions	Connecting socially and reaching out	*We like face-to-face, we like paper, we like all of that, we’ve got to have, but, actually, we also need this other dimension, which is both about trying to get broader engagement, but, also, about how we present ourselves to the world. (Sarah)*
	Delivering online and offline information	*You can go on the website and you can find one there, but actually, have one delivered to you too. (Iris)*

**Table 3:** Themes, example codes, and data from the thematic analysis exploring digital audio.

Themes	Example Code	Data
Engaging citizens	Reaching different age groups	*Why is it* [that people] *are not interested? I’m sure they are but we’re certainly not reaching them. The younger cohort. People in their 60s. (Sarah)*
	Inviting public feedback	*For the moment, everything is very hidden. I put this out and wait for people to tell us what they think... (Iris)*
Demonstrating impact	Creating digital evidence	Digital technologies are a creative way of evidencing work that had been done. (Author 1, field notes)
	Tracking audience engagement	Engaging digitally offers the opportunity to diagnose trends and be more responsive to the audience. (Author 1, field notes)
